# The development of an internet-based outpatient cardiac rehabilitation intervention: a Delphi study

**DOI:** 10.1186/1471-2261-10-27

**Published:** 2010-06-10

**Authors:** Corneel Vandelanotte, Trudy Dwyer, Anetta Van Itallie, Christine Hanley, W Kerry Mummery

**Affiliations:** 1 Centre for Physical Activity Studies, Institute for Health and Social Sciences Research, Central Queensland University, Rockhampton 4702, Australia

## Abstract

**Background:**

Face-to-face outpatient cardiac rehabilitation (OCR) programs are an important and effective component in the management of cardiovascular disease. However, these programs have low participation rates, especially among patients who live rural or remote. Hence, there is a need to develop OCR programs that provide an alternative to face-to-face contact such as by using the Internet. Only a very limited number of Internet-based OCR programs have been developed and evaluated. Therefore, the purpose of this study was to identify issues that are relevant to the development of an Internet-based OCR intervention.

**Methods:**

A three-round Delphi study among cardiac rehabilitation experts was conducted. In the first round, 43 experts outlined opinions they had on the development of an online ORC platform into an open-ended electronic questionnaire. In the second round, 42 experts completed a structured (five-point scale) electronic questionnaire based on first round results, in which they scored items on their relevance. In the third round, the same experts were asked to re-rate the same items after feedback was given about the group median relevance score to establish a level of consensus.

**Results:**

After the third round, high consensus was reached in 120 of 162 (74%) questionnaire items, of which 93 (57% of 162 items) also had high relevance according to the experts. The results indicate that experts strongly agreed on desired website content, data obtained from the patient, and level of interaction with patients that should be part of an Internet-based OCR intervention.

**Conclusion:**

The high rates of consensus and relevance observed among cardiac rehabilitation experts are an indication that they perceived the development and implementation of an Internet-based ORC intervention as feasible, and as a valuable alternative to face-to-face programs. In many ways the experts indicated that an Internet-based ORC program should mimic a traditional face-to-face program, and emphasize the crucial role of the cardiac rehabilitation manager who interacts with patients from a distance. The present study revealed practical insights into how Internet OCR interventions should be designed and opens the door for the development of such an intervention to be subsequently examined in a longitudinal and experimental study.

## Background

In developed countries cardiovascular disease (CVD) continues to be the leading cause for mortality and morbidity in men and women and reducing its burden remains an important public health priority [1,2]. Controlling the CVD epidemic requires a multifaceted strategy targeting recognised modifiable risk factors, aimed at both the general population and high-risk individuals (primary prevention), as well as aimed at individuals with established CVD or recovering from an cardiovascular event (secondary prevention) [3]. People who have previously suffered from a cardiovascular event, such as a cerebrovascular accident (stroke) or a myocardial infarction (heart attack), are at elevated risk of having further cardiac events [4,5]. As a result cardiac rehabilitation programs are recognised as an important component of the rehabilitation process and essential in the prevention of future cardiac events [3,4,6].

There is overwhelming evidence that completion of cardiac rehabilitation programs can reduce distress, disability, increase confidence, enhance risk factor modification and reduce recurrence rates [3,4,6]. More specifically, there is strong evidence that individuals who participate in an outpatient cardiac rehabilitation (OCR) program following hospital discharge (also called phase II rehabilitation) can significantly improve functional capacity, quality of life, social and psychological health, and provide a five-year survival advantage of up to 35% [5,7]. However, despite documented evidence of the benefits of OCR programs referral, attendance and completion of OCR programs is problematic [7-10]. Studies have shown alarmingly low rates of enrolment and participation into phase II cardiac rehabilitation programs, with numbers ranging between 10% and 30% of eligible patients [5,11]. Specifically for Australia, participation rates for OCR programs range between 20% and 40% of completion, with significantly lower rates for patients from rural and remote areas [12,13].

Many reasons for these low participation rates have been reported, such as patients not being referred, being too busy, having to return to work and/or not perceiving the OCR program as important [8,14]. However, one of the foremost barriers to participation is distance to, and lack of facilities to participate in OCR sessions, with high (transport) costs as an associated barrier [11,15,16]. This is especially relevant for people living in rural and remote communities. Countries such as Australia, Canada and the United States are typified for having considerable rural and remote populations that have limited access to health care services such as provided to those living in the urban centres, resulting in a geographical inequity of care [11]. The need for alternatives to the traditional face-to-face delivery of OCR programs is emphasised by the observation that rates of cardiovascular disease are higher among people living rural and remote areas [2,3]. The use of new telecommunication technologies has been suggested as a potential solution to extend the reach of health care practitioners and connect with patients from distance [11,17-19].

Increased use and accessibility of the internet has brought about many opportunities for individuals to access innovative Internet-based health promotion programs at their convenience from home [20,21]. Previous studies examining a vast array of issues within the health promotion field (such as smoking cessation, nutrition, physical activity, depression and diabetes management) have successfully demonstrated the use and applicability of website-delivered interventions to improve health behaviours [22-26]. However, according to a systematic review evaluating telemedicine interventions for coronary heart disease, only a very limited number of Internet-based OCR programs have been developed and evaluated [27]. Two Internet interventions were identified, one of which had only 13 patients [9,11]. Both interventions showed promising results towards the viability of Internet-based OCR interventions. Southard et al. [9] showed a decrease in cardiovascular events in patients who received the intervention, and concluded that the intervention was very cost-effective even though only a limited number of patients participated in the intervention. Zutz et al. [11] showed significant improvements in cholesterol levels, triglycerides, physical activity and self-efficacy in the intervention group. Despite these studies very little is known about how to best tailor and deliver an interactive Internet-based OCR intervention, that can also be accessed by people living in rural and remote areas.

The current study is part of a larger research project aimed at the development, implementation and evaluation of an Internet-based OCR program (eOCR), and the results of this study will be used to guide the development of the new OCR intervention. Hence, the aims of this study were: firstly, to identify issues that are important to the development of a Internet-based OCR intervention and secondly, to reach consensus among cardiac rehabilitation experts about importance and relevance of these issues. More specifically, issues regarding desired website content, information that needs to be collected from patients, and the level of interaction between cardiac case managers and patients using the online platform needed to be explored in depth to allow the development of the new OCR intervention.

## Methods

**Figure 1:**
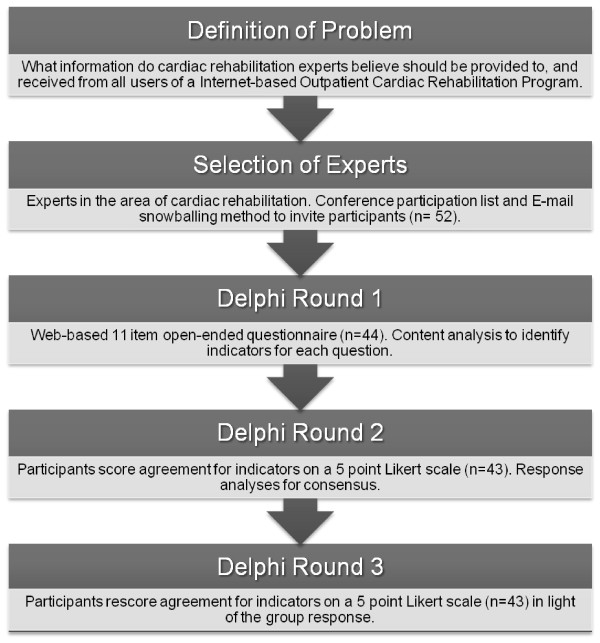
**Delphi Study Overview**.

A three-round Delphi study was conducted using web-based questionnaires. In a Delphi study a panel of experts is consulted over several rounds. It is a systematic approach which aims to engage a large number of experts in a process to derive consensus in a group on a topic where the required information is incomplete or scarcely available [28]. In the past, rehabilitation researchers have also applied the Delphi technique in order to gain consensus about unexplored issues [29-31]. The Delphi methodology was chosen for this study for several reasons. Firstly, the main characteristics of a Delphi study - anonymity, iteration, controlled feedback and statistical group response - allow participants to give and change (after receiving feedback) their opinion freely [32-34]. Secondly, this method is particularly suited for generating ideas about topics on which the scientific knowledge is scarce. And finally, the Delphi technique is convenient in situations where face-to-face discussions are impractical, when for example the experts are geographically dispersed, as a Delphi study can be completed using the Internet [35]. The use of the Internet is also helpful as it ensures anonymity of the experts and it allows the experts to complete each questionnaire at their own convenience. The first round of a Delphi study is aimed at identifying which factors are important in relation to the topic of interest (Figure 1). In the second round the aim is to determine the relative importance of each of the factors identified in the first round. The third round is conducted to achieve consensus on the importance of the items identified in the first round. All the questions in this three round Delphi study were pre-tested among experts in the field of cardiac-rehabilitation research; feedback was sought on readability, word ordering, understand-ability and question order effects, modifications were made when needed. The entire Delphi study was carried out within 3 months (September to November 2007). Prior to the commencement of the study ethical clearance was obtained from the Human Research Ethics Committee of the Central Queensland University.

### First Round

#### Procedures and Participants

For the first round experts in the field of cardiac rehabilitation were selected. As cardiac rehabilitation programs are delivered by a multidisciplinary health care team it was decided that the panel of experts should reflect this diversity of disciplines, including cardiac rehabilitation nurses, cardiac rehabilitation coordinators, physicians, exercise physiologists, allied heath workers and academics that specialise in intervention research, cardiac rehabilitation and website-delivered health behaviour change. The selected experts needed to have every day experience working with phase II cardiac rehabilitation programs and be willing and able to express opinions about the subject. Professionals attending the 2007 Australian Cardiovascular Health and Rehabilitation Association (ACRA) conference were invited to participate in the study, as well as cardiac rehabilitation professionals known by the research team. These individuals were sent an information letter via e-mail outlining the purpose of the Delphi study, inviting them to participate and recommend other suitable cardiac rehabilitation professionals. All eligible participants received access to an online consent form on a password protected website and were informed that clicking on the link would be accepted as their consent to participate in the study.

All the cardiac rehabilitation specialists who were invited to participate in the study were asked to participate in all three rounds of the Delphi study. Participants received a reminder one week after first being invited to access the website and complete the questionnaire that was part of the first round of the Delphi study, a second reminder was send another week later. In total, participants had three weeks to complete the first-round questionnaire. All data were collected by means of a web-based questionnaire, which was developed and administered through the Population Research Laboratory at Central Queensland University.

#### Questionnaire

A web-based questionnaire consisting of 11 open ended questions was used in the first round. This questionnaire, developed specifically for this study, was asking about the opinions of cardiac rehabilitation professionals on the development of an online cardiac rehabilitation platform and was taking into account three groups of users the application: (i) the patient who is undertaking the rehabilitation; (ii) the support groups members, who are the spouses, partners, families or friends of the patient, and (iii) the case managers, who are the cardiac rehabilitation specialists who will manage the patient by distance using the internet-based platform. An example of a question is 'What do you believe are the key areas of information that should be presented to the patient?'. The first round questionnaire was primarily conducted to provide input for the second round questionnaire.

#### Data analyses

Responses were analysed using content analysis [36], which included identifying similar words, phrases and quotations, that were grouped, coded, listed and counted. Answers that described the same variable were summarised into one item. Three researchers did this separately and independently and differences in interpretations were discussed. The analysis resulted in a list of items used to build the second-round questionnaire.

### Second Round

#### Procedures and Participants

The same participants of Round One were invited to participate in the second round. They received an e-mail with a hyperlink to complete the second round questionnaire. After one week non-responders received a first e-mail reminder, and they received a second and final reminder another week later.

#### Questionnaire

For the second round, a structured 162-item web-based questionnaire was developed. The questionnaire still had the same original 11 questions as in Round One. However, rather than being open ended, each question now had a number of closed items (ranging from 10 to 30 items per question) that needed to be rated. Participants had to indicate to what extent they agreed with the statements about the Internet-based OCR platform on five point Likert scales (strongly agree, agree, neutral, disagree, strongly disagree). An example of a question is: 'How do you think information should be presented on the website to the patients?' Examples of items (each of which has to be rated separately) related to this question are: 'as static text', 'as a video animation', or 'as a PowerPoint presentation'. Forty-eight items related to the patient; 45 items related to the support group members; and 69 items related to the cardiac case managers.

#### Data analysis

Two methods are generally used in Delphi studies to summarize the extent to which participants agree with the importance of the factor under consideration: the median score and the interquartile range [37]. The median score, defined as the score that falls exactly in the middle of a group of scores, also referred to as the 50^th ^percentile score, was calculated for each item to score the agreement on the relevance or importance of each item. The interquartile range (IQR) score was calculated to asses the extent of agreement between experts about the scored relevance [37,38]. The IQR represents the distance between the 25^th ^and the 75^th ^percentile values, with smaller values indicating a higher degree of consensus. An IQR score of 2 means that 50% of all the scores fall within two points on the scale. On a five point Likert scale an IQR of 2 can be considered as moderate consensus, an IQR of 1 as good consensus, and an IQR of 0 as high consensus, as it is the highest level achievable using this method [32]. The distinction between importance and consensus was made since it is possible that there is high consensus (Low IQR) among experts that an item is not important (low median score).

### Third Round

#### Procedures and Participants

All the experts who completed the second-round questionnaire were invited for the third and final round. The same procedures as in round two were applied.

#### Questionnaire

In the third round, participants received an adapted version of the second-round web-based questionnaire. In this round the questionnaire displayed second-round group results for each item. Underneath each rating option (going from one to five) it showed the percent of participants who had selected it in the previous round. For example: 65% of participants 'agreed' with the item in the previous round, and 23% of participants 'strongly agreed' with the statement. Further, according to Delphi methodology, items on which high consensus was obtained in the second round (IQR = 0) were excluded from the third round questionnaire [28,32]. 137 out of 162 items were retained for the third-round questionnaire. Participants were asked to rate the items again, now having been exposed to the feedback of their peers.

#### Data analysis

Again the group median ratings and IRQ were calculated to determine the level of agreement as to the relevance of each item [37,38]. Items that had a median relevance score of 5 (highest) and that had an IQR of 0 (lowest) were deemed as essential to the development of the web-based OCR platform. SPSS version 15.0 was used for the analyses.

## Results

### Participants

In the first round 52 people, who were deemed eligible and willing to participate out of 115 invitees, were invited into the study (Figure 1). Forty-four of them completed the online first-round questionnaire and came from a variety of backgrounds, including cardiac rehabilitation coordinators and managers (n = 17), cardiac rehabilitation nurses (n = 13), cardiac researchers (n = 6), allied health workers (n = 5) and physicians involved in cardiac rehabilitation (n = 3). Two people withdrew and six did not complete the first-round questionnaire. Forty-three people participating in the first round also completed second- and third-round questionnaires.

### Numeric Outcomes

#### First Round

Since the responses of the cardiac rehabilitation experts in the first round were used to inform the second round, the results of the first round are shown as the items for each question in the table (Additional file 1). This table lists all the items that were found to be important in the content analysis. In total, the content analysis resulted in 162 items being retained for rating in subsequent rounds. These items related to 11 open ended questions on the original first-round questionnaire and three different user groups (patients, support group members, and cardiac case managers).

#### Second Round

The results of the second round are shown in The table (Additional file 1). High consensus (IQR = 0) was reached on 25 items during this round. In 22 of these items the relevance or importance was also high (Median = 5). Twelve items did not have good consensus (IQR > 1) after round 2.

#### Third Round

The results of the third round are also shown in The table (Additional file 1). Overall, there was an improvement in consensus (expressed as a lower IQR score) in 102 (62%) items when going from the second to the third round. High consensus was reached in an additional 95 items, of which 71 also had a high relevance. Hence, in total high consensus was reached in 120 out of 162 (74%) items, of which 93 had high relevance (57% of 162 items). There was good (IQR = 1) or high consensus on all items after round three.

### Relevance and Consensus

Very high scores for relevance and consensus were reported for items that asked about what information should be collected from the patient via the online platform (Q3 in The table (Additional file 1)), as well as for items that asked about what information that should be available for the cardiac case manager using the system (Q9 in The table (Additional file 1)); respectively 87% and 93% of these items had and IQR of 0. The only items that scored lower on relevance for these questions related to whether or not pedometer step counts (as an expression of activity levels) should be collected from patients, and whether the cardiac case manager should have the ability to interact with patients and support group members via an online forum. The consensus was considerably lower for items relating to what information should be collected from support group members (Q7 in The table (Additional file 1)), only 6 out of 13 (46%) items scored very high on relevance and consensus.

Questions relating to *what *information that should be presented to both patients (Q1 in Table 1) and support group members (Q5 in The table (Additional file 1)) scored moderately high, with 60% and 70% of the items scoring high on relevance and consensus respectively. Items relating to presenting information about cardiac rehabilitation, self management, behaviour change, individual progress scored high on both relevance and consensus. Items relating to presenting information about the health care system and testimonials from other patients or support group members scored lower.

Questions relating to *how *information should be collected from and presented to both patients (Q2 and Q4 in The table (Additional file 1)) and support group members (Q6 and Q8 in The table (Additional file 1)) scored relatively high on consensus, but much lower in terms of relevance. For these items, the number of items that scored high on both relevance and consensus was low and ranged between 0 and 30%. The lowest relevance was reported for the use of an online forum, PowerPoint presentations and incentives; the highest relevance was reported for the use of individual tailoring, images and diagrams and frequently asked questions.

Fairly high consensus and relevance was observed for items related to what the cardiac case manager should be able to do in the system (Q10 in The table (Additional file 1)); 15 out of 21 items scored high on both consensus and relevance (71%). Items related to the patient (e.g. provide direction and feedback, monitor progress, enter goals) scored higher compared to items related to the website itself (e.g. upload documents, customise patient access, recommend websites, reply to forum postings). When asked what else the program should do (Q11 in The table (Additional file 1)) 13 out of 18 (72%) items scored high on both relevance and consensus. Highest scoring items were 'addressing legal and confidentiality issues', 'follow best practice guidelines', 'use validated measures' and 'allow evaluation'. Lowest scoring items were 'provide paper-based resources in addition to the website', 'provide diaries' and 'replicate a community feel'.

## Discussion

Despite their effectiveness face-to-face OCR programs have very low participation rates [5,11], especially among patients who live in rural and remote communities. Internet-based OCR programs might provide a viable alternative, but there is a lack of such interventions [27]. The aim of this study was to identify what issues are important to the development of an Internet-based OCR intervention and to reach consensus among cardiac rehabilitation experts about importance and relevance of these issues. Despite the heterogeneous sample of experts, the results of this study showed a high rate of consensus (74%) and relevance (57%). A strong improvement in level of consensus was observed between the second and third round of the study. This is comparable to what was found in other Delphi studies [33,39,40], and implies that experts strongly agreed about the desired website content, data obtained from the patient via the Internet, and level of interaction between patient and cardiac case manager that should be part of an Internet-based OCR intervention.

The results in The table (Additional file 1) show that the experts had a strong and well defined knowledge of *what *information should be collected from the patient for use in the online OCR program, as well as *what *information should be available from the online system for the cardiac case manager. Consensus was lower in relation to what information should be collected from the support group members. This is most likely because traditional face-to-face OCR programs predominantly focus on the patient only and less on support group members, as it is more difficult to integrate them into a face-to-face program, whereas that is not the case for Internet-delivered interventions [23,24]. Nonetheless, experts emphasised the importance of social support for patients during their recovery and that support group members should be part of an Internet OCR program. Further, lower relevance and importance levels were also observed for items relating to *how *information should be collected from and presented to patients and support group members. This is likely because participants in the study were experts in cardiac-rehabilitation and not so much in website design and functionality, and thus might have a lower understanding of what is required and functional in an Internet-delivered intervention [17]. Nevertheless, consensus on these topics was very high.

The results indicate that a cardiac case manager should assume a similar role in an Internet-based OCR program to that of the cardiac rehabilitation coordinator in a traditional face-to-face program. The experts strongly agreed that the role of the cardiac case manager would be pivotal to the conduct of the program, and as such would regularly be interacting with both the patient, the patients support group members and where necessary the patients' general practitioner. This is in line with the study of Southard et al. [9], where patients were recommended to log on to the website on a weekly basis to communicate with the cardiac case manager. Within this context, this study identified that issues of confidentiality and security of patient information were considered as essential by the experts. In relation to the content of the program, experts strongly agreed with the idea that the patient should have access to and be provided with all information consistent with a standard face-to-face OCR program, and that the program should be consistent with established best practice guidelines in the area of cardiac rehabilitation. Experts stressed the importance of individual tailoring inside the Internet-delivered OCR program and indicated that it should also provide patients with tools and applications to set goals and improve risk factors for cardiovascular disease through behavioural self-management. All these elements are in line with many preconceived notions with regard to internet delivered interventions in other fields of health promotion research [22-26].

Some limitations of this Delphi study must be mentioned. First, the respondents may not be representative for 'all' experts on cardiac rehabilitation, as they were mostly Australian and relatively low in number. Also, further selection bias might have occurred, as recruitment to be part of the study partially depended on attending a conference and having access to e-mail. Secondly, as mentioned before, the cardiac rehabilitation experts in the study might not have had sufficient knowledge about website design and functionality to answer a number of specific questions ('how to' questions). Thirdly, the use of the Delphi-method in itself has limitations: its time consuming and demanding for participants, opinions were equally weighted regardless of level of experience of the cardiac rehabilitation experts, and participants did not have the option to justify their opinions in the second and third round of data collection, which makes the handling of divergent opinions difficult. And fourthly, no input was sought from cardiac rehabilitation patients themselves, nor from professionals dealing with psychosocial aspects of the recovery process, such as psychologists or social workers; their views might have strengthened the outcomes of this Delphi study.

## Conclusions

Based on the knowledge and experience of experts in the field, this Delphi study revealed practical insights that are important in the development of an Internet-based OCR program. The high rates of consensus and relevance observed among cardiac rehabilitation experts are an indication that they perceived the development and implementation of an Internet-based ORC intervention as feasible, needed and as a valuable alternative for face-to-face programs. Experts have well defined opinions on what information should be collected from patients, as well as what information should be available to them. In many ways the experts indicated that an Internet-based ORC program should mimic a traditional face-to-face program, and emphasize the crucial role of the cardiac rehabilitation manager who interacts with patients from a distance.

It would have been complex, if not impossible, to derive these insights from the literature, as few OCR programs have been evaluated to date, and none of them have provided any insight as to what elements and issues are important in the development of such programs. However, this knowledge is crucial to improve public health impact of OCR interventions. The next logical step is to develop an Internet-based OCR intervention based on the outcomes of this study and evaluate its efficacy and effectiveness in longitudinal, experimental and controlled studies. Within this context it will also be important to evaluate how effective cardiac rehabilitation managers are in integrating the use of an online OCR platform into their daily practice, as well as whether their need for ongoing guidance and training to use it to its full benefit.

## Competing interests

The authors declare that they have no competing interests.

## Authors' contributions

CV analysed the data, interpreted the results and wrote the manuscript. TD was involved in conception and design of the study, drafted sections of the manuscript and revised drafts of the manuscript. AVI was involved in conception and design of the study, data collection, interpretation of results, and revised drafts of the manuscript. CH was responsible for data collection and revised drafts of the manuscript. WKM was involved in conception and design of the study, interpretation of results and revised drafts of the manuscript. All authors read and approved the final manuscript.

## Pre-publication history

The pre-publication history for this paper can be accessed here:

http://www.biomedcentral.com/1471-2261/10/27/prepub

## Supplementary Material

Additional File 1 **Table**. This file contains the Table which gives a description for second and third round Delphi study results. Click here for file
