# Supplementary material for: The development of an internet-based outpatient cardiac rehabilitation intervention: a Delphi study
Source: BMC Cardiovasc Disord. 2010 Jun 10;10:27. doi: 10.1186/1471-2261-10-27 (PMC2891620; doi:10.1186/1471-2261-10-27)
Supplement: Additional File 1 — Table. This file contains the Table which gives a description for second and third round Delphi study results. [file 1471-2261-10-27-S1.DOC]

**Table I** – Results of the Delphi study for the second (N = 43) and third round (N = 43).

| **Item** | **Second Round** | | | **Third Round** | | | **Item** | **Second Round** | | | **Third Round** | | |
| --- | --- | --- | --- | --- | --- | --- | --- | --- | --- | --- | --- | --- | --- |
|  | **X** | **Mdn** | **IQR** | **X** | **Mdn** | **IQR** |  | **X** | **Mdn** | **IQR** | **X** | **Mdn** | **IQR** |
| **Q1. What do you believe are the key areas of information that should be presented to the patient?** |  |  |  |  |  |  | **Q8. How would this information best be collected from the support group members?** |  |  |  |  |  |  |
| 1. Cardiac rehabilitation | 4.81 | 5 | 0 | – | – | – | 1. Contact forms | 3.86 | 4 | 1 | 3.86 | 4 | 0 |
| 2. Behaviour change management | 4.84 | 5 | 0 | – | – | – | 2. E-mails | 4.16 | 4 | 1 | 4.07 | 4 | 0 |
| 3. Self-management skills | 4.88 | 5 | 0 | – | – | – | 3. Forum postings | 3.51 | 3 | 1 | 3.44 | 3 | 1 |
| 4. Importance of interaction with their doctors | 4.65 | 5 | 1 | 4.70 | 5 | 1 | 4. Questionnaires | 3.95 | 4 | 0 | – | – | – |
| 5. Importance of interaction with health professionals | 4.49 | 5 | 1 | 4.65 | 5 | 1 | 5. Diary | 3.60 | 4 | 1 | 3.77 | 4 | 0 |
| 6. Healthcare system | 3.84 | 4 | 2 | 3.67 | 4 | 1 | 6. Tick-flick | 4.02 | 4 | 1 | 4.02 | 4 | 0 |
| 7. Referrals to specific health care professionals | 4.47 | 5 | 1 | 4.63 | 5 | 0 | 7. Written/open-ended questions | 3.67 | 4 | 1 | 3.77 | 4 | 0 |
| 8. Their case manager's contact details | 4.51 | 5 | 1 | 4.88 | 5 | 0 | 8. Closed/scaled questions | 3.79 | 4 | 1 | 3.77 | 4 | 0 |
| 9. Their individual progress | 4.74 | 5 | 0 | – | – | – | 9. Numeric entry | 3.77 | 4 | 1 | 3.95 | 4 | 0 |
| 10. Testimonials from other patients | 3.81 | 4 | 1 | 3.44 | 4 | 1 | 10. Drop-down boxes | 3.93 | 4 | 2 | 4.02 | 4 | 0 |
| **Q2. How do you think this information should be presented to the patient when using online delivery?** |  |  |  |  |  |  | **Q9. What information should be available from the online system for the case manager?** |  |  |  |  |  |  |
| 1. Static text | 4.19 | 4 | 1 | 4.09 | 4 | 0 | 1. Contact details of all rehabilitation team members | 4.74 | 5 | 0 | – | – | – |
| 2. Audio | 4.44 | 4 | 1 | 4.44 | 4 | 1 | 2. Patients' referral information | 4.81 | 5 | 0 | – | – | – |
| 3. Podcasts | 3.77 | 4 | 1 | 3.63 | 4 | 1 | 3. Program summary data | 4.70 | 5 | 1 | 4.93 | 5 | 0 |
| 4. Video/animation | 4.56 | 5 | 1 | 4.65 | 5 | 1 | 4. Professional forum for case managers | 4.44 | 5 | 1 | 4.79 | 5 | 0 |
| 5. Powerpoint | 3.56 | 3 | 1 | 3.49 | 3 | 1 | 5. Patient login and program usage statistics | 4.42 | 5 | 1 | 4.81 | 5 | 0 |
| 6. Interactive quiz/questionnaire | 4.26 | 4 | 1 | 4.19 | 4 | 1 | 6. Patient risk factors | 4.81 | 5 | 0 | – | – | – |
| 7. Interactive question and answer | 4.37 | 4 | 1 | 4.56 | 5 | 1 | 7. Patient activities of daily living | 4.60 | 5 | 1 | 4.77 | 5 | 0 |
| 8. Images/diagrams | 4.60 | 5 | 1 | 4.81 | 5 | 0 | 8. Patient mood states/affect states | 4.79 | 5 | 0 | – | – | – |
| 9. Frequently asked questions | 4.47 | 5 | 1 | 4.79 | 5 | 0 | 9. Patient demographics | 4.65 | 5 | 1 | 4.88 | 5 | 0 |
| 10. Downloadable documents (pdf, word) | 4.12 | 4 | 2 | 4.16 | 4 | 1 | 10. Patient contact details | 4.79 | 5 | 0 | – | – | – |
| 11. Small incentives (certificates etc) | 3.79 | 4 | 2 | 3.70 | 4 | 1 | 11. Patient baseline assessment | 4.77 | 5 | 0 | – | – | – |
| 12. Individual Tailoring | 4.42 | 5 | 1 | 4.72 | 5 | 0 | 12. Patient family history | 4.58 | 5 | 1 | 4.84 | 5 | 0 |
| **Q3. What info should be collected from the patient?** |  |  |  |  |  |  | 13. Patient cardiologist contact details | 4.63 | 5 | 1 | 4.86 | 5 | 0 |
| 1. Diet/nutrition | 4.81 | 5 | 0 | – | – | – | 14. Patient GP contact details | 4.65 | 5 | 1 | 4.88 | 5 | 0 |
| 2. Alcohol | 4.63 | 5 | 1 | 4.72 | 5 | 1 | 15. Patient other health professionals contact details | 4.42 | 5 | 1 | 4.84 | 5 | 0 |
| 3. Activities of daily living | 4.58 | 5 | 1 | 4.86 | 5 | 0 | 16. Patient co-morbidities and other conditions | 4.79 | 5 | 1 | 4.91 | 5 | 0 |
| 4. Physical activity | 4.93 | 5 | 0 | – | – | – | 17. Patient medications | 4.81 | 5 | 0 | – | – | – |
| 5. Exercise | 4.74 | 5 | 0 | – | – | – | 18. Patient GP management plan | 4.53 | 5 | 1 | 4.81 | 5 | 0 |
| 6. Step counts | 3.93 | 4 | 1 | 3.58 | 4 | 1 | 19. Number of hospital admissions for patient | 4.28 | 4 | 1 | 4.72 | 5 | 0 |
| 7. Blood pressure | 4.65 | 5 | 1 | 4.81 | 5 | 0 | 20. Patient symptoms | 4.79 | 5 | 0 | – | – | – |
| 8. Weight | 4.70 | 5 | 1 | 4.86 | 5 | 0 | 21. Patient clinical information | 4.74 | 5 | 1 | 4.91 | 5 | 0 |
| 9. Blood sugar levels (for diabetics) | 4.72 | 5 | 1 | 4.93 | 5 | 0 | 22. Patient diagnosis | 4.86 | 5 | 0 | – | – | – |
| 10. Smoking | 4.81 | 5 | 0 | – | – | – | 23. Patient recovery progress and information | 4.67 | 5 | 1 | 4.91 | 5 | 0 |
| 11. Symptoms | 4.77 | 5 | 0 | – | – | – | 24. Patient rehabilitation goals | 4.74 | 5 | 1 | 4.91 | 5 | 0 |
| 12. Mood states/affect states | 4.77 | 5 | 0 | – | – | – | 25. Patient management action plans | 4.70 | 5 | 1 | 4.88 | 5 | 0 |
| 13. Quality of life | 4.47 | 5 | 1 | 4.56 | 5 | 0 | 26. Info and support provided to patient so far | 4.60 | 5 | 1 | 4.86 | 5 | 0 |
| 14. Rehabilitation goals | 4.72 | 5 | 0 | – | – | – | 27. Patients' perception of individual issues | 4.56 | 5 | 1 | 4.84 | 5 | 0 |
| 15. Questions to rehabilitation group | 4.47 | 5 | 1 | 4.74 | 5 | 0 | 28. Patient knowledge | 4.58 | 5 | 1 | 4.91 | 5 | 0 |
| 16. Questions to case managers | 4.60 | 5 | 1 | 4.91 | 5 | 0 | 29. Patient interaction with forum | 4.23 | 4 | 1 | 4.26 | 4 | 1 |
| **Q4. How do you think this information would best be** |  |  |  |  |  |  | 30. Support group members' interaction with forum | 4.19 | 4 | 1 | 4.02 | 4 | 1 |
| **collected from the patient?** |  |  |  |  |  |  | **Q10. What should the case manager be able to** |  |  |  |  |  |  |
| 1. Contact forms | 3.86 | 4 | 2 | 3.79 | 4 | 1 | **do into the system?** |  |  |  |  |  |  |
| 2. E-mails | 4.14 | 4 | 1 | 4.12 | 4 | 0 | 1. Customise what the patient sees | 4.00 | 4 | 2 | 4.09 | 4 | 1 |
| 3. Forum postings | 3.53 | 4 | 1 | 3.60 | 4 | 1 | 2. Control the release of information to patients | 3.84 | 4 | 2 | 4.00 | 4 | 0 |
| 4. Questionnaires | 4.07 | 4 | 0 | – | – | – | 3. Enter clinical information/updates | 4.70 | 5 | 1 | 4.91 | 5 | 0 |
| 5. Diary | 4.14 | 4 | 1 | 4.12 | 4 | 0 | 4. Enter baseline and follow-up assessment data | 4.70 | 5 | 1 | 4.91 | 5 | 0 |
| 6. Tick-flick | 4.19 | 4 | 1 | 4.05 | 4 | 0 | 5. Provide direction to patient | 4.77 | 5 | 0 | – | – | – |
| 7. Written/open-ended questions | 3.58 | 4 | 1 | 3.81 | 4 | 0 | 6. Enter rehabilitation goals for patient | 4.53 | 5 | 1 | 4.70 | 5 | 0 |
| 8. Closed/scaled questions | 3.63 | 4 | 1 | 3.70 | 4 | 1 | 7. Monitor progress of patient | 4.74 | 5 | 1 | 4.88 | 5 | 0 |
| 9. Numeric entry | 3.93 | 4 | 0 | – | – | – | 8. Provide feedback to patient | 4.74 | 5 | 1 | 4.88 | 5 | 0 |
| 10. Drop-down boxes | 4.16 | 4 | 1 | 4.09 | 4 | 0 | 9. Monitor self-management and care plans | 4.67 | 5 | 1 | 4.88 | 5 | 0 |
| **Q5. What are the key areas of information that should** |  |  |  |  |  |  | 10. Monitor patient medications | 4.56 | 5 | 1 | 4.74 | 5 | 0 |
| **be presented to the support group members?** |  |  |  |  |  |  | 11. Upload existing documents (pdf, word) | 4.47 | 5 | 1 | 4.58 | 4 | 1 |
| 1. Cardiac rehabilitation | 4.77 | 5 | 0 | – | – | – | 12. Recommend websites | 4.33 | 4 | 1 | 4.42 | 4 | 1 |
| 2. Behaviour change management | 4.63 | 5 | 1 | 4.95 | 5 | 0 | 13. Make corrections to entries made by patients | 3.86 | 4 | 2 | 4.02 | 4 | 1 |
| 3. Self-management | 4.63 | 5 | 1 | 4.91 | 5 | 0 | 14. Provide case manager contact details | 4.58 | 5 | 1 | 4.77 | 5 | 0 |
| 4. Expectations from self during rehabilitation process | 4.56 | 5 | 1 | 4.91 | 5 | 0 | 15. Provide emergency action information | 4.72 | 5 | 1 | 4.77 | 5 | 0 |
| 5. Expectations from patient during rehabilitation process | 4.60 | 5 | 1 | 4.88 | 5 | 0 | 16. View GP contact details | 4.29 | 5 | 1 | 4.79 | 5 | 0 |
| 6. Myth clarifications | 4.60 | 5 | 1 | 4.84 | 5 | 0 | 17. Provide reports to patients | 4.60 | 5 | 1 | 4.86 | 5 | 0 |
| 7. Case manager's contact details | 4.42 | 5 | 1 | 4.81 | 5 | 0 | 18. Provide replies to forums | 4.47 | 5 | 1 | 4.65 | 5 | 1 |
| 8. The patient's progress | 4.09 | 4 | 1 | 3.93 | 4 | 0 | 19. Communicate, contact and interact with patient | 4.72 | 5 | 1 | 4.88 | 5 | 0 |
| 9. Testimonials from other patients | 3.65 | 4 | 1 | 3.60 | 4 | 1 | 20. Contact other case managers in the program | 4.40 | 5 | 1 | 4.70 | 5 | 0 |
| 10. Testimonials from other support group members | 3.98 | 4 | 2 | 3.95 | 4 | 0 | 21. Update the website with new information | 4.40 | 5 | 1 | 4.60 | 5 | 0 |
| **Q6. How should this information be presented to the support group members when using online delivery?** |  |  |  |  |  |  | **Q11. Overall the online program should…** |  |  |  |  |  |  |
|  |  |  |  |  |  |  | 1. Provide telephone support in addition | 4.56 | 5 | 1 | 4.77 | 5 | 0 |
| 1. Static text | 4.02 | 4 | 2 | 3.91 | 4 | 0 | 2. Provide recorded diaries to then be entered online | 4.02 | 4 | 1 | 4.14 | 4 | 1 |
| 2. Audio | 4.33 | 4 | 1 | 4.53 | 5 | 1 | 3. Provide paper-based resources in addition | 4.12 | 4 | 1 | 4.14 | 4 | 1 |
| 3. Pod-casts | 3.84 | 4 | 2 | 3.67 | 4 | 1 | 4. Include a list of referral services | 4.51 | 4 | 1 | 4.84 | 5 | 0 |
| 4. Video/animation | 4.37 | 4 | 1 | 4.72 | 5 | 1 | 5. Provide cues for case manager to assist patients | 4.42 | 4 | 1 | 4.81 | 5 | 0 |
| 5. PowerPoint | 3.63 | 4 | 1 | 3.51 | 3 | 1 | 6. Encourage the sharing of information (forums etc) | 4.44 | 4 | 1 | 4.65 | 5 | 1 |
| 6. Interactive quiz/questionnaire | 4.02 | 4 | 2 | 4.14 | 4 | 0 | 7. Provide an aesthetically pleasing site | 4.60 | 5 | 1 | 4.79 | 5 | 0 |
| 7. Interactive question and answer | 4.28 | 4 | 1 | 4.28 | 4 | 1 | 8. Replicate a community feel | 4.16 | 4 | 1 | 4.14 | 4 | 1 |
| 8. Images/diagrams | 4.42 | 4 | 1 | 4.65 | 5 | 1 | 9. Be provided in a variety of languages | 4.63 | 5 | 1 | 4.81 | 5 | 0 |
| 9. Frequently asked questions | 4.56 | 5 | 1 | 4.79 | 5 | 0 | 10. Provide quality external website links | 4.49 | 5 | 1 | 4.72 | 5 | 1 |
| 10. Downloadable documents (pdf, word) | 4.12 | 4 | 1 | 4.09 | 4 | 0 | 11. Address legal and confidentiality issues | 4.60 | 5 | 1 | 4.91 | 5 | 0 |
| 11. Small incentives (certificates etc) | 3.21 | 3 | 1 | 3.23 | 3 | 0 | 12. Address duty of care requirements of manager | 4.56 | 5 | 1 | 4.88 | 5 | 0 |
| 12. A patient-controlled release designed for support group | 4.41 | 4 | 1 | 3.74 | 4 | 0 | 13. Use recognised measures to collect information | 4.72 | 5 | 1 | 4.91 | 5 | 0 |
| **Q7. What information do you think should be collected from the support group members?** |  |  |  |  |  |  | 14. Provide optional more in-depth information | 4.56 | 5 | 1 | 4.88 | 5 | 0 |
|  |  |  |  |  |  |  | 15. Allow for a maintenance phase online | 4.49 | 5 | 1 | 4.72 | 5 | 0 |
| 1. Their demographics | 4.07 | 4 | 1 | 4.09 | 4 | 0 | 16. Allow for the integration of clinical information | 4.51 | 5 | 1 | 4.79 | 5 | 0 |
| 2. Their relationship to patient | 4.58 | 5 | 1 | 4.86 | 5 | 0 | 17. Follow best practice guidelines | 4.81 | 5 | 1 | 4.91 | 5 | 0 |
| 3. Their contact details | 4.33 | 4 | 1 | 4.47 | 5 | 1 | 18. Allow for the evaluation of the program | 4.77 | 5 | 1 | 4.93 | 5 | 0 |
| 4. Their overall health | 4.02 | 4 | 1 | 4.00 | 4 | 0 |  |  |  |  |  |  |  |
| 5. Their coping and managing since the cardiac event | 4.53 | 5 | 1 | 4.91 | 5 | 0 |  |  |  |  |  |  |  |
| 6. Their quality of life | 4.21 | 4 | 1 | 4.26 | 4 | 1 |  |  |  |  |  |  |  |
| 7. What information helps most when supporting patient | 4.63 | 5 | 1 | 4.93 | 5 | 0 |  |  |  |  |  |  |  |
| 8. Expectations of themselves as support members | 4.49 | 4 | 1 | 4.47 | 5 | 1 |  |  |  |  |  |  |  |
| 9. Any support, services they are accessing | 4.47 | 4 | 1 | 4.67 | 5 | 1 |  |  |  |  |  |  |  |
| 10. Affects of the cardiac event on their home life | 4.67 | 5 | 1 | 4.91 | 5 | 0 |  |  |  |  |  |  |  |
| 11. Recovery issues of patient | 4.49 | 5 | 1 | 4.86 | 5 | 0 |  |  |  |  |  |  |  |
| 12. The progress of the patient | 4.42 | 4 | 1 | 4.65 | 5 | 1 |  |  |  |  |  |  |  |
| 13. Questions regarding the patients' rehabilitation | 4.60 | 5 | 1 | 4.84 | 5 | 0 |  |  |  |  |  |  |  |

Note: - some item descriptions were shortened in order to present a clear and easy to overview table.

- X = Average score, Mdn = Median Score (range 1 to 5), IQR = Interquartile Range (range 4 to
